# De novo variant of *SETD1A* causes neurodevelopmental disorder with dysmorphic facies: A case report

**DOI:** 10.1111/pcn.13310

**Published:** 2021-12-03

**Authors:** Jia Zhang, Qiuji Tao, Zuozhen Yang, Yang Li, Jing Gan

**Affiliations:** ^1^ Department of Pediatrics West China Second University Hospital, Sichuan University Chengdu China; ^2^ Key Laboratory of Obstetrics & Gynecologic and Pediatric Diseases and Birth Defects of the Ministry of Education Sichuan University Chengdu China; ^3^ Cipher Gene LLC Beijing China

A 7‐year‐old female was born after a full‐term pregnancy. Enlarged ventricles (0.7–1.0 cm) were observed during pregnancy without hydrocephalus. Large head circumference (39 cm), hypotonia, facial hemangioma, patent foramen ovale, and hypothyroidism were found after birth. The Bayley Scales of Infant and Toddler Development, Second Edition (BSID‐II), at about 11 months of age revealed a motor development index <50, the development level equivalent to a 6‐month‐old infant. The cognitive development index was 64, equivalent to a 7‐month‐old infant. Head magnetic resonance imaging (MRI) suggested bilateral white matter dysplasia and ventriculomegaly (Supplement Fig. S1A–D). Chromosome karyotype analysis, copy number variations, and screening of congenital metabolic disorders showed no abnormalities. When the child was 3 years old, generalized tonic‐clonic seizures occurred. Video electroencephalogram (VEEG) revealed mild background slowing (Supplement Fig. S1F, G). Her seizures were effectively controlled by levetiracetam, and there were no seizures for 4 years. Two days before admission, her epilepsy reemerged. An emergency blood glucose test revealed a blood glucose level of 1.3 mmol/L but returned to normal levels after receiving glucose supplementation. Physical examination showed a height of 114 cm (1 SD–2 SD), a weight of 21.5 kg (P25), and a head circumference of 58.5 cm (>3 SD). She displayed specific abnormal facial features of macrocephaly, high forehead, low nose bridge, inverted nostril, thick lips, and a thin face. Additionally, her facial and physical features act asymmetrically, left limbs were smaller on the left than the right side, head deviation to the left, torticollis, right‐eye strabismus, short and thick fingers, flat feet, soft soles, and deformed feet (obvious on right foot: middle toe bent inward, right fourth toe short, parallel to the fifth toe) (Supplement Fig. S2A). The sternum is slightly valgus with mild hypotonia and hypertrichosis. Interictal arterial spin labeling MRI (Supplement Fig. S1E) displayed relative hyperperfusion in multiple areas of the left brain and left cerebellar. Repeated VEEG showed no obvious abnormality.

A de novo variant in *SETD1A* (SET domain‐containing protein 1A) was detected [NM_014712.3: exon8: c.2120_2121insA (p.Gly708Argfs*117)] caused by an insertion between 708th and 709th amino acid resulting in a truncated protein via early termination. Sanger sequencing confirmed the variant in her family (Supplement Fig. S2B). The variant was not detected in public databases and classified as pathogenic according to the American College of Medical Genetics and Genomics guidelines (Supplement Table S1). Other pathogenic variants of genes known to be associated with development, epilepsy, or intellectual disability were not found in the proband.

According to the clinical manifestations, *SETD1A* gene mutation, and previous literature reports, the girl was diagnosed with *SETD1A*‐related neurodevelopmental disorder with dysmorphic facies. She started rehabilitation training at 1 year old, and her language development was fair. The patient was followed up with for 6 months. Slightly uncoordinated movement and posture as well as poor balance were observed. However, her cognitive level continued to improve (Wechsler Intelligence Scale for Children [WISC] score of 65). Currently, she is enrolled in kindergarten and her verbal memory is good. She can communicate normally with slightly slower reaction times and poor logical thinking. Recently, there was a short attention span and poor control of urine and feces, but no feeding difficulties.

*SETD1A* is a member of the COMPASS (complex proteins associated with Set1) family of proteins, all of which have H3K4 methyltransferase activity and are closely related to neural development. *SETD1A* has been identified as a risk gene for schizophrenia, and individuals with *SETD1A* variants may define a new subgroup of schizophrenia, often associated with obsessive‐compulsive disorder.^1^ Here, we described a proband with a de novo variant in *SETD1A* [NM_014712.3:exon8: c.2120_2121insA (p.Gly708Argfs*117)] identified by whole‐exome sequencing. The main manifestations of the proband were moderate global developmental delay, epilepsy, hypotonia, short stature, special facial features, hemangioma, toe deformity, white matter dysplasia, and ventricular dilation. Several studies have shown that individuals with *SETD1A* variants may have unique characteristics, including epilepsy, general developmental delay, and minor facial deformities (Table 1).^2^, ^3^, ^4^

**Table 1:** Analysis of pathogenicity of variants in *SETD1A*

Phenotype	Overall	Truncating	Splice	Missense
Developmental retardation/mental retardation	100% (26 of 26)	100% (4 of 4)	100% (3/3)	100% (4/4)
Mental/behavior abnormalities	75% (18 of 24)	75% (3 of 4)	67% (2/3)	N
Facial deformity	42% (11 of 26)	75% (3 of 4)	67% (2/3)	N
Hypotonia	42% (11 of 26)	25% (1 of 4)	33% (1/3)	N
Epilepsy	38% (10 of 26)	50% (2 of 4)	N	100% (4/4)
Musculoskeletal abnormalities	32% (8 of 25)	25% (1 of 4)	N	N
Short stature	29% (7 of 24)	50% (2 of 4)	N	N
Hemangioma	15% (4 of 26)	50% (2 of 4)	67% (2/3)	N
Digit deformity	15% (4 of 26)	25% (1 of 4)	N	N
Head and neck deflection	12% (3 of 26)	25% (1 of 4)	N	N
Skeleton deformity	12% (3 of 26)	25% (1 of 4)	N	N
Macrocephaly*	4% (our case)	25% (1 of 4)	N	N
Facial and limb asymmetry*	4% (our case)	25% (1 of 4)	N	N
Stubby fingers*	4% (our case)	25% (1 of 4)	N	N
Hypertrichosis*	4% (our case)	25% (1 of 4)	N	N

#Kummeling et al summarized 15 children with SETD1A variants, but the corresponding relationship between genotype and phenotype of each patient was not shown in his literature. Therefore, mutation information does not include these 15 patients. #Our case contains all of these phenotypic features.

* These novel phenotypes were exclusively in our case.

Compared with previous reports, our case showed novel phenotypes such as macrocephaly, hypertrichosis, stubby fingers, and unique face and toe deformities, namely, the facial features, trunk, and limbs on the left side were relatively smaller than that on the right. In addition, our patient had no obvious speech development disorder or behavioral abnormality. Levetiracetam is effective in the treatment of seizures in our case. In addition, there was another report that showed phenobarbital to improve SETD1A‐related epilepsy.^4^ This case enriches our understanding of *SETD1A*‐related neurodevelopmental disorders, expanding the phenotype and genotype spectrum. Through literature review, we also found that the truncating variants were more severe in clinical phenotypes, most of which were accompanied by mental and behavioral disorders, facial deformities, and short stature. This case provides valuable information for clinical diagnosis and genetic counseling.

## Disclosure statement

The authors have no competing interests to declare. Jia Zhang and Qiuji Tao contributed equally to this work.

## Ethics approval and consent to participate

The ethics committee of West China Second University Hospital judged that there was no need to review this case.

## Consent for publication

Written informed consent was obtained from her parents for the publication of this case report.

## Availability of data and materials

All data generated or analyzed during this study are included in this published article.

## Supporting information


**Figure S1**. Head magnetic resonance imaging suggested bilateral white matter dysplasia and ventriculomegaly (Fig. 1A, 1B, 1C, 1D). Video electroencephalography revealed mild background slowing (Fig. 1F, 1G)

**Figure S2**. The patient's facial and physical features act asymmetrically, left limbs smaller on the left than the right side, head deviation to the left, torticollis, right‐eye strabismus, short and thick fingers, flat feet, soft soles, and deformed feet (Fig. 2A). Pedigree and Sanger sequencing of the family confirmed the variant in her family (Fig. 2B).

**Table S1**. Phenotype related to variant type.
