# Supplementary material for: De novo variant of SETD1A causes neurodevelopmental disorder with dysmorphic facies: A case report
Source: Psychiatry Clin Neurosci. 2021 Dec 3;76(2):58–9. doi: 10.1111/pcn.13310 (PMC9300109; doi:10.1111/pcn.13310)
Supplement: Supplementary file 1 — Figure S1. Head magnetic resonance imaging suggested bilateral white matter dysplasia and ventriculomegaly (Fig. 1A, 1B, 1C, 1D). Video electroencephalography revealed mild background slowing (Fig. 1F, 1G) [file PCN-76-58-s003.pptx]

## Slide 1
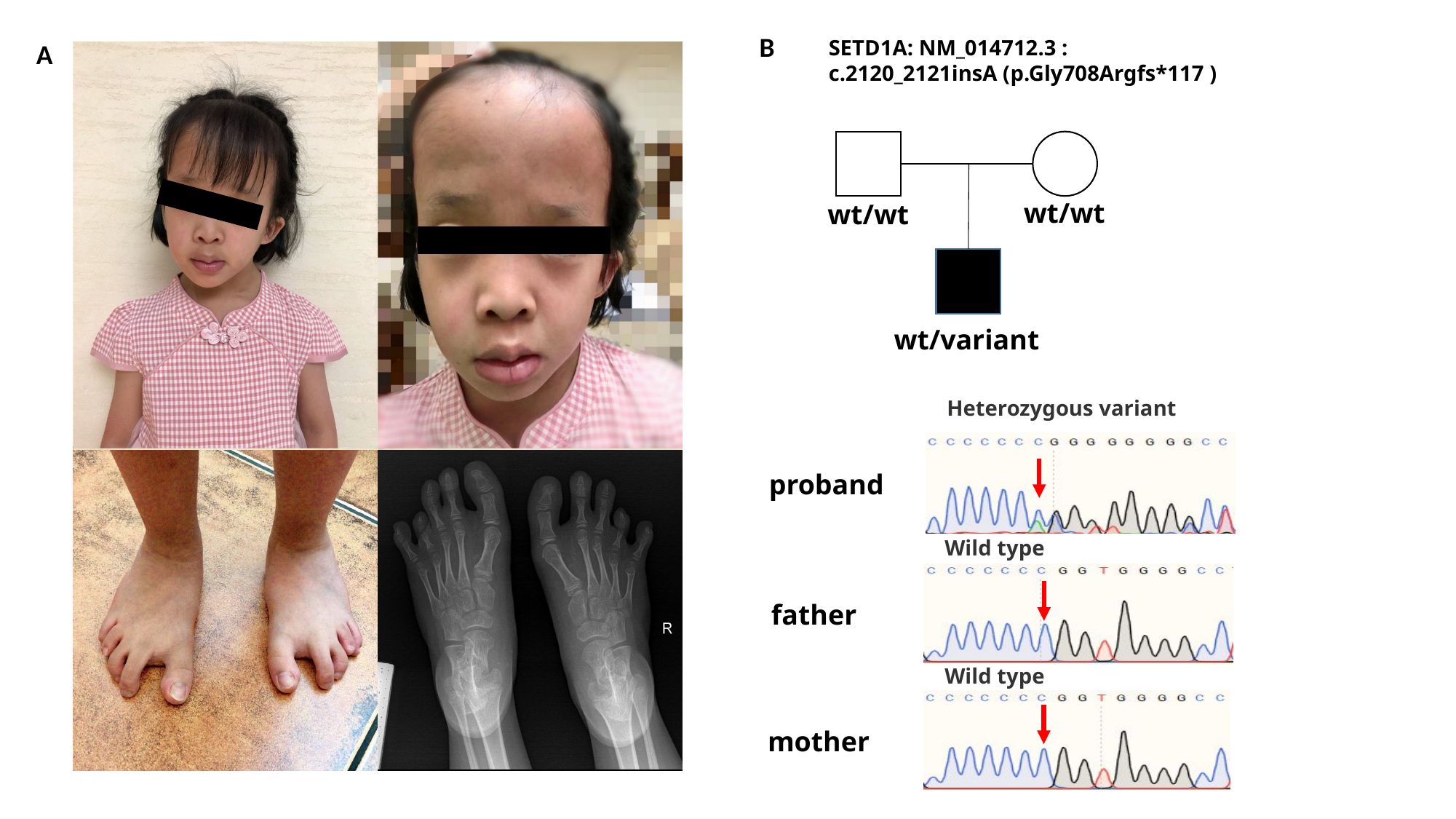

B
SETD1A: NM_014712.3 :
c.2120_2121insA (p.Gly708Argfs*117 )
wt/wt
wt/wt
wt/variant
A
Heterozygous variant
proband
Wild type
father
Wild type
mother
