# Supplementary figures and images for: De novo variant of SETD1A causes neurodevelopmental disorder with dysmorphic facies: A case report
Source: Psychiatry Clin Neurosci. 2021 Dec 3;76(2):58–9. doi: 10.1111/pcn.13310 (PMC9300109; doi:10.1111/pcn.13310)

## Slide 1
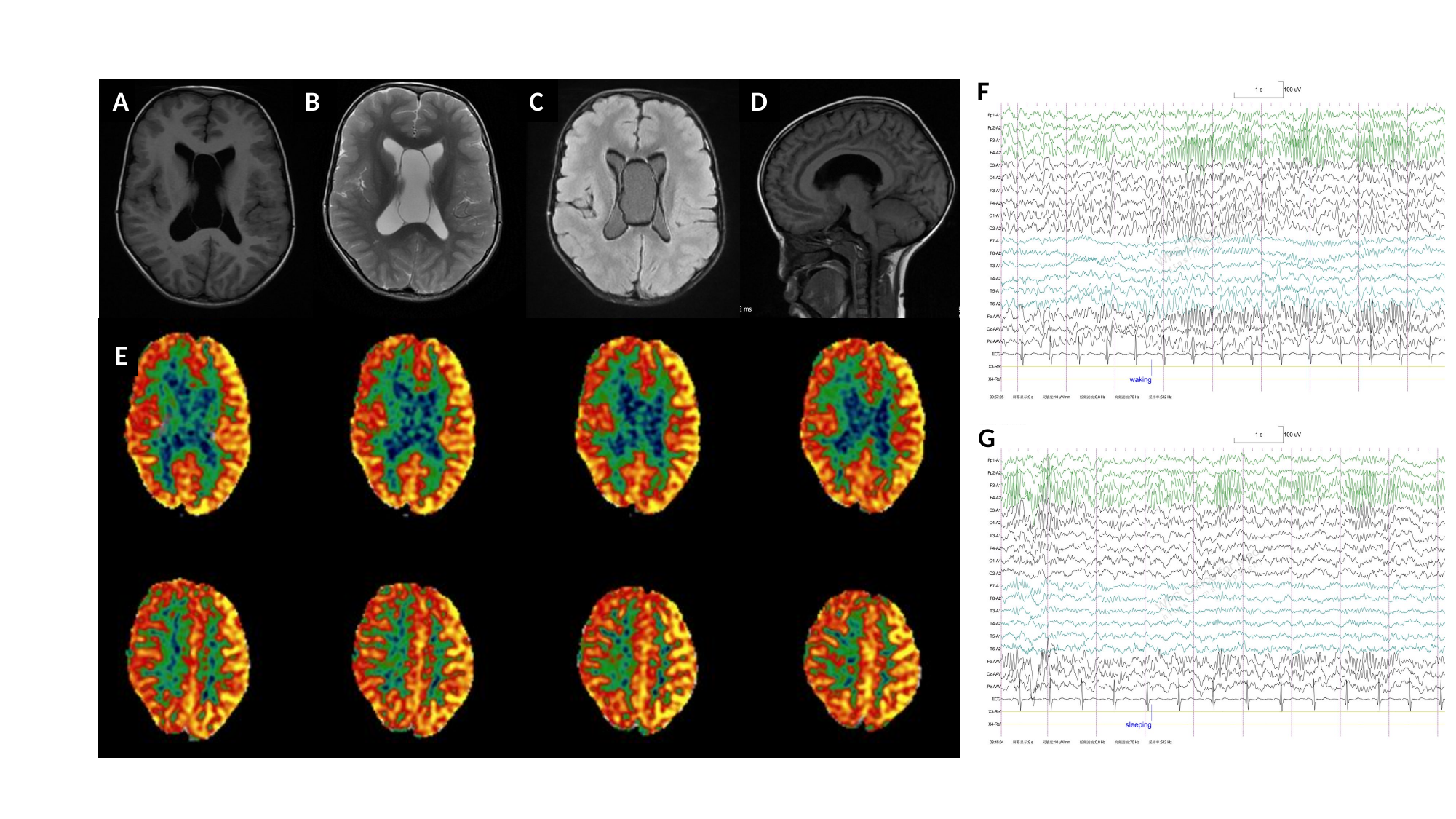

A
B
C
D
E
F
F
G
E
G

Supplement: Supplementary file 2 — Figure S2. The patient's facial and physical features act asymmetrically, left limbs smaller on the left than the right side, head deviation to the left, torticollis, right‐eye strabismus, short and thick fingers, flat feet, soft soles, and deformed feet (Fig. 2A). Pedigree and Sanger sequencing of the family confirmed the variant in her family (Fig. 2B). [file PCN-76-58-s002.pptx]
