# Supplementary material for: De novo variant of SETD1A causes neurodevelopmental disorder with dysmorphic facies: A case report
Source: Psychiatry Clin Neurosci. 2021 Dec 3;76(2):58–9. doi: 10.1111/pcn.13310 (PMC9300109; doi:10.1111/pcn.13310)
Supplement: Supplementary file 3 — Table S1. Phenotype related to variant type. [file PCN-76-58-s001.docx]

**Supplement Table 1**

Phenotype related to variant type.

| Gene | Variant | Inheritance | MAF | | | Evidence | Category |
| --- | --- | --- | --- | --- | --- | --- | --- |
|  |  |  | ExAc | gnomAD | 1000 genome |  |  |
|  |  |  |  |  |  |  |  |
| *SETD1A* | c.2120_2121insA (p.Gly708Argfs*117) | De novo | NE | NE | NE | PVS1+PS2+PM2_supporting | Pathogenic |

Transcript: NM_014712.3; MAF: minor allele frequency, NE: not exist
